# Zootherapy as traditional therapeutic strategy in the Cholistan desert of Bahawalpur‐Pakistan

**DOI:** 10.1002/vms3.491

**Published:** 2021-05-03

**Authors:** Saeed Ahmad, Muhammad Akram, Muhammad Riaz, Naveed Munir, Imtiaz Mahmood Tahir, Hina Anwar, Rabia Zahid, Muhammad Daniyal, Faiza Jabeen, Ejaz Ashraf, Ghulam Sarwar, Ghulam Rasool, Syed Muhammad Ali Shah

**Affiliations:** ^1^ College of Agriculture, University of Sargodha Sargodha Pakistan; ^2^ Department of Eastern Medicine Government College University Faisalabad Faisalabad Pakistan; ^3^ Department of Allied Health Sciences Sargodha Medical College University of Sargodha Sargodha Pakistan; ^4^ Department of Biomedical Lab Sciences, School of Health Sciences University of Management and Technology Lahore Pakistan; ^5^ College of Allied Health Professional Government College University Faisalabad Faisalabad Pakistan; ^6^ TCM and Ethnomedicine Innovation & Development International Laboratory School of Pharmacy Hunan University of Chinese Medicine Changsha China; ^7^ Department of Zoology Government College University Faisalabad Faisalabad Pakistan

**Keywords:** bioactive constituents, Cholistan, faunistic resources, folk medicine, medicinal animal, Pakistan

## Abstract

The use of traditional medicines has tremendously increased over the past few decades. Approximately 80% of the world's population relies on traditional medicines for their primary healthcare needs because of their cost effectiveness and efficiency with no or minimal side effects. Zootherapy refers to the use of medicines that are prepared or derived from animals or from their products. The current study documented the folk knowledge related to the practice of various animal‐derived products and ethnozoological based drugs used as medicines by the residents of the Cholistan desert of Bahawalpur (Pakistan). In this regard 46 knowledgeable and reliable elderly people, hakims and spiritual healers ranging from 35–60 years of age having knowledge related to zootherapy were included in the current study. A field survey from February 2006 to November 2007 was conducted by interviewing the selected respondents through a structured questionnaire. They provided knowledge regarding the use of animals and their derived products in traditional medicine. The zootherapeutic knowledge was based on both domestic animals as well as wild animals. A total of 20 animal species were included in the study, among which nine animals were domestic while 11 were wild animals. Among selected animals, nine were mammals, four birds, four reptiles and three insects. It was reported that camel was the most commonly used (*n* = 32 respondents) among mammals while Pigeon (*n* = 39 respondents), Spiny‐tailed lizard (*n* = 41 respondents) and Indian honey bee (*n* = 27 respondents) among birds, reptiles and insects, respectively, have significant use for the treatment of different diseases. Based on this communication we could recommend that this type of abandoned knowledge should be considered for the management and conservation of faunistic resources. However, the advantageous role of animals and their products was reported but more extensive research is required to explore the bioactive constituents in the raw material of these animals responsible for their beneficial effects.

## Graphical abstract

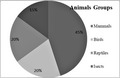

## 1 INTRODUCTION

World Health Organization (WHO) estimates that the majority (approximately 80%) of the world's population rely mainly on plant and animal‐based medicine. Literature reports indicated that traditional medicine accounts for around 40% of all health care medicines in China while 65% of Indian population living in rural areas uses traditional medicines to meet their primary health care needs. Traditional medicines are becoming more popular in underdeveloped and developed countries as well and being used by 48% population in Australia, 49% in France, 70% in Canada and 42% in the United States of America (Mahawar & Jaroli, 2007; Rakhmanina and Anker, 2011). Since ancient time, in different cultures of the world, animals and their products obtained from various parts are used for medicinal purposes (Lev, 2003) and such practices still exist in folk medicine. In modern civilizations, among many other well‐known therapies practiced worldwide, zootherapy constitutes a significant substitute. Body parts of domestic and wild animals (e.g., tusks, feathers, skins, bones and hooves) and their by‐products (e.g., fat) form the principal constituents in the formulation of curative, protective and preventive medicine (Adeola, 1992; Angeletti et al., 1992; Solavan et al., 2004). In Traditional Chinese Medicine more than 1,500 animal species have been documented for their medicinal uses. Of the 252 essential chemicals that have been recorded by the WHO, 11.1% from plants, while 8.7% come from animals. In the United States of America (USA) among 150 prescriptions medicine that is currently in use, 27 drugs have an animal origin. In India, approximately 15%–20% of the Ayurvedic medicines are obtained from animals. In Unani system of medicines nearly 200 drugs derived from the animal source are documented which are claimed to be useful for the management of different diseases. In Pakistan, 31 substances (constituting 9% of all the medicinal substances) were recorded in the inventory of folk medicines (Mahawar & Jaroli, 2007).

**FIGURE 1:**
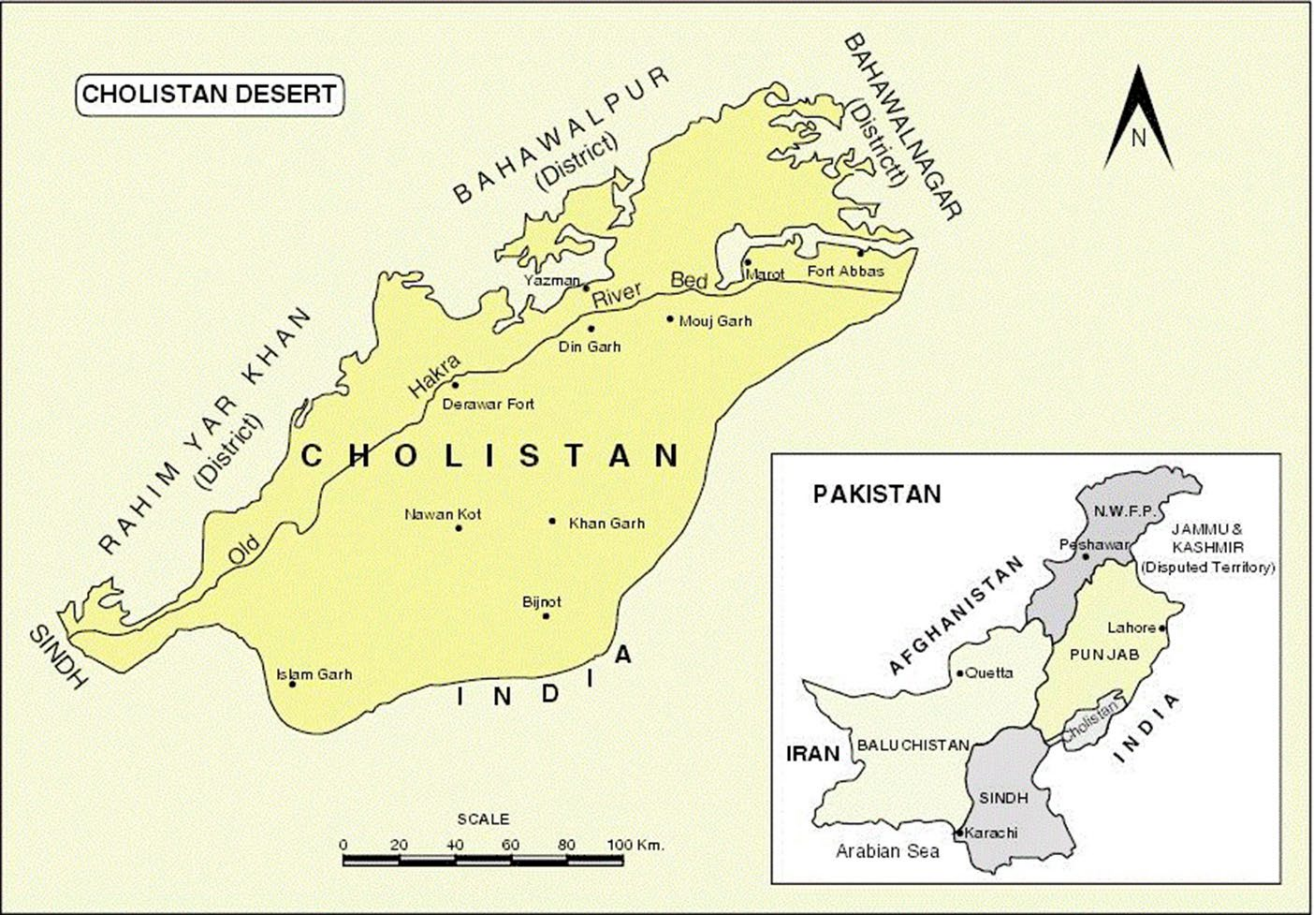
Map of Pakistan presenting five provinces (one occupied Jammu Kashmir) with associated countries and the Cholistan desert

Cholistan desert is uniquely located wild land of its own kind with the scarcity of endemic flora and fauna. The Cholistan desert is situated in the south‐west of Punjab (Pakistan) occupies an area of 16,000 km^2^ with highly saline soils and brackish subsoil aquifer (Figure 1). A human population of 110,000 herders depends exclusively upon livestock for their livelihood including sheep, goats, cattle, camels and rarely donkeys. Life sustainability revolves around annual precipitation ranging from 100 to 250 mm per annum. The water harvested from run‐off is collected in low‐lying areas called ‘Toba’ which is the only source of potable water. The water is consumed from the same ‘Toba’ both by inhabitants and animals until it is exhausted, a situation which compels them to migrate to another water source ‘Toba’, ‘kund’ (well or canal area) a basic reason for their nomadic way of life (Akbar et al., 1996; Arshad et al.,^2006^, ^2007^).

In Cholistan desert summer is extremely harsh and punishing. It experiences soaring temperature touching 52°C or more with periodical long droughts (Mughal, 1997). Zebu/humped cattle (*Bos Taurus indicus* Linnaeus, 1758), camel *(Camelus dromedaries* Linnaeus, 1758), goat (*Capra hircus* Linnaeus, 1758) and sheep (*Ovisaries* Linnaeus, 1758.) are domestic animals used in daily life. The main wild animals found in the area are rabbits (*Oryctolagus cuniculus* Linnaeus, 1758), deer (*Antilope cervicapra* F. Cuvier, 1823), sand lizards (*Lacerta agilis* Linnaeus, 1758), crow (*Corvus splendens* Vieillot, 1817), crow pheasant or greater coucal (*Centropus sinensis* Stephens, 1815), cochineal insect (*Dactylopius coccus* Costa, 1835), sanda (*Saara hardwickii* Gray 1827), Indian/Spectacled Cobra (*Naja oxiana* Eichwald, 1831), desert monitor (*Varanus griseus* Daudin, 1803) and carpenter ants (*Camponotus* spp.) are commonly used by local people as traditional medicine for the treatment of wide range of disorders or clinical conditions. For majority of the people in Cholistan desert, traditional medicine in fact, remains the major or only source of health care. The health care practices begin at the household levels, where the elder family member, man or women, possess a great deal of information on the diagnosis and treatment of common ailments. This unique combination of local knowledge is practiced by the local healer. The treatments are given by these local practitioners throughout the Cholistan desert and its adjoining areas who cure the diseases with various drugs of animal origin. Arshad et al. (2002) has given comprehensive knowledge about the use of medicinal plants in the Cholistan desert (Arshad et al., 2002). However, animals which are being put to therapeutic practice in the area yet to be emphasized. So, the current survey‐based study was planned to investigate the zootherapeutic aspects of Cholistan desert used for the treatment and survival of the local population with limited resources.

## 2 MATERIAL AND METHOD

### 2.1 Selection of study sites and respondents

The Cholistan desert is situated in the south‐west of Punjab (Pakistan) occupies an area of 16,000 km^2^ with highly saline soils and brackish subsoil aquifer (Figure 1). Darawar fort, Din Ghar, Mouj Ghar, Khan Ghar, Nawan Kot, Islam Ghar and Bijnot are the major areas of the Cholistan desert which were visited to collect the information regarding zootherapy. These sites were purposively selected based on the availability of many traditional healers, the presence of different ethnic groups, and the accessibility of the area. To record the first‐hand knowledge about ethnozoological uses of animals of the Cholistan desert and its adjoining areas were explored from February 2006 to November 2007 through various field expeditions. In this regard 46 knowledgeable and reliable elderly people, hakims, and spiritual healers from selected regions were contacted. The selection of respondents was made on their recognition as knowledgeable members concerning traditional medicine. Informed consent was taken from the respondents whether they know the use of animals in the healing practices. Generally, they have information regarding plant‐based remedies, but they also know some use of animals in therapeutics.

### 2.2 Sampling and data collection

We asked the method of formulation, its administration or application, and the proper dose of the remedy. According to them, they acquired the knowledge of traditional medicines primarily through parental heritage, or because they have practiced about the medicinal significance of animal to treat themselves or their relatives. The ethnozoological data was collected through questionnaires, interviews and focus group discussion with selected residents of different areas of Cholistan and purposively, 46 key respondents were selected.

### 2.3 Informant consensus

During the study, each informant visited three times in order to confirm the reliability of the ethnozoological information. Consequently, the responses of an informant that was not in harmony with each other were rejected since they were considered as unreliable information.

### 2.4 Animal identifications

**FIGURE 2:**
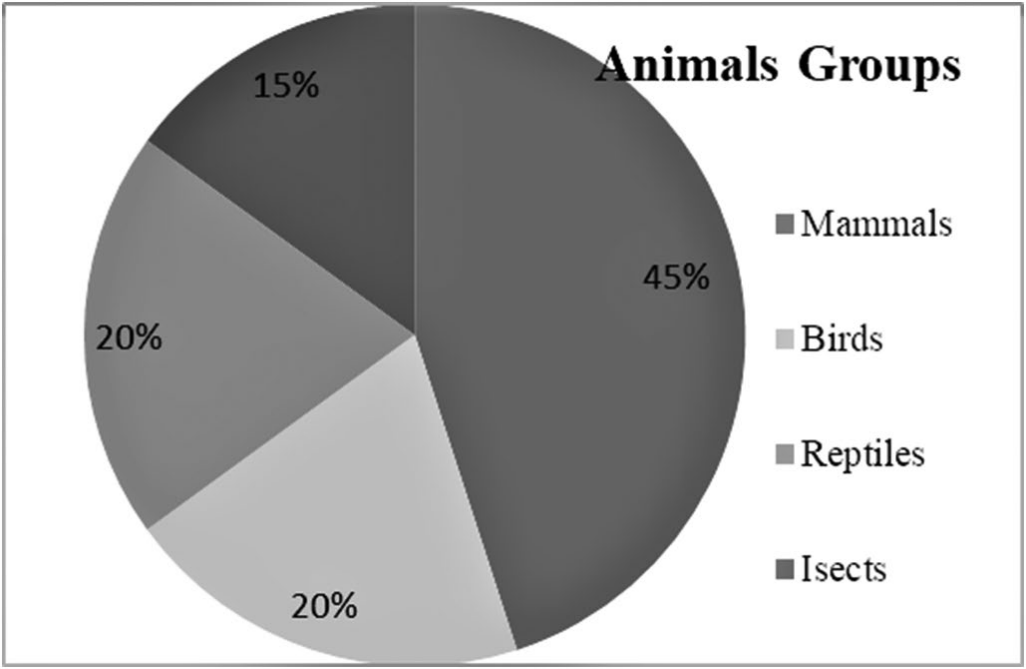
Percentage of major four animal groups used as a zootherapeutic treatment for different ailments in the Cholistan desert of Bahawalpur‐Pakistan

A total of 20 different medicinal animal species were documented that are used for various ethno‐medicinal purposes. Among the selected medicinal animals, 9 were domestic animals while 11 were wild animals. All the nine domestic animals were mammals while wild animals included four birds, four reptiles and three insects (Figure 2 and Table 1). Animal species and their scientific names were identified and confirmed by Local University in Bahawalpur‐Pakistan. In spite of their significance, as compared to plants, studies on the therapeutic usages of animals and their body parts have been neglected. It is important to highlight that some folk medicinal systems, like the Traditional Chinese Medicine, is approved by WHO and accepted by one fourth of the world human population.

**TABLE 1:** Age and gender‐wise distribution of the questionnaires to local informants

Age group	Gender	No of Questionnaires
Hakims	Male	11
	Female	None
Old people	Male	41
	Female	21
Total	73	

## 3 RESULTS

Collected data from indigenous people practicing traditional medicine for many decades explored that oral route, massaged and direct applying to wounds were the major routes of administration. No injectable route is being practiced till now in selected areas. The most commonly mentioned species were spiny‐tailed lizard (*n* = 41), pigeon (*n* = 39), camel (*n* = 32), buffalo (*n* = 29), honeybee (*n* = 27), goat (*n* = 21) and sheep (*n* = 19). The most important parts or products of animals used as folk medicine are milk, meat, wool, fat, egg, fresh blood, gall bladder, butter, nector, horns, liver, flesh and whole animal. There are different methods for preparation of different dosage forms and the summary is presented in Table 2. Further, the respondents informed that animal‐based traditional medicines have therapeutic potential to treat different diseased conditions like general weakness, asthma, epilepsy, skin disease, jaundice, rickets, tuberculosis, conjunctivitis, sexual debility, paralysis, cough, meningitis, piles, impotency, leucoderma, diabetes, leprosyand arthritis. Further, it was reported from 29 informants that zebu is used for weakness, asthma, epilepsy and skin diseases, 32 informants reported that camels are used for jaundice, skin diseases and rickets, 21 informants reported the use of goats for tuberculosis and conjunctivitis, 19 informants reported the use of sheep for general and sexual debility and skin wounds. Given to newborn babies in diluted form, 21 informants reported that buffalos are used for general weakness and paralysis, 15 informants reported the use of chicken for weakness and cough, 24 informants reported the use of donkey for asthma and tuberculosis, 39 informants reported that pigeon is used for paralysis, tuberculosis and asthma. Whereas 14 informants reported that milk of women is used for meningitis, 27 informants reported the use of honey for weakness and eye diseases. A total of 17 informants reported the use of black buck for asthma, tuberculosis, weakness, and piles; 26 informants reported that rabbits are used for asthma and tuberculosis while 25 informants reported the use of sand lizard for impotency and sexual debility, 3 informants reported the use of house crow for leucoderma, 17 informants reported the use of crow pheasant for asthma and diabetes, 22 informants reported that Indian or spectacled cobra is used for leprosy, leucoderma and arthritis, 19 informants reported that geyr's spiny‐tailed lizards are used for sexual organ debility in males and breast weakness in females, arthritis and paralysis. Twenty‐seven informants reported the use of ant for muscular weakness, 27 informants reported the use of desert monitor for arthritis and muscular weakness. This zootherapeutic knowledge was based on both domestic as well as wild animals and the detailed information is presented in Table 2.

**TABLE 2:** Use of animals and their products in folk medicine by the people of Cholistan desert of Bahawalpur, Pakistan

S.No.	Local name	English name	Scientific name	No. of informants reporting the use	Parts used	Ailments	Method of preparation	Mode of administration
1	*Makoray*	Ant	*Camponotus* sp.	18	Whole insects	Muscular weakness	The whole animal is burnt in cow's ghee and oil is used as medicine.	External
2	*Bair bahooti*	Cochineal insect	*Dactylopius* sp.	19	Whole animal	Cough, sexual organ debility	The insect is dried powdered and taken in cough, while burnt in oil and this oil is massaged in sexual organ debility.	Orally and externally
3	*Nag*	Indian/ Spectacled Cobra	*Naja oxiana*	37	Fat	Leprosy. leucoderma, and arthritis	Fat is obtained from the animal and massaged on the affected part of the skin.	Externally
4	*Sandha*	Spiny‐tailed lizard	*Saara hardwickii*	41	Fat	Sexual organ debility in males, and breast weakness in females. arthritis and paralysis.	Fat is obtained by the incision to the animal.	External
5	*Raig mahi*	Sand lizard	*Ophiomorus* sp.	25	Whole animal	Impotency, sexual debility	The animal is dried and powdered and taken orally for impotency while it is cooked in cows ghee and massaged on sexual organ for weakness	Orally and Externally
6	*Goh*	Desert monitor	*Varanus griseus*	19	Fat	Arthritis and muscular weakness	Fat is obtained by the incision to animal	External
7	*Makhi*	Indian Honey bee	*Apis indica*	27	Nector	Weakness, eye diseases	Nector is obtained and orally taken while in ophthalmic diseases applied in eyes.	Orally and Externally
8	*Kawa*	House Crow	*Corvus splendens*	17	Flesh	Leucoderma	The flesh of the crow is cooked in mustard oil and applied on the skin	Externally
9	*Greater Coucal*	Crow pheasant	*Centropus sinensis*	21	Flesh	Asthma and diabetes	Cooked flesh is used in asthma and diabetes	Orally
10	*Kabooter*	Pigeon	*Columba livia*	39	Fresh blood and Meat	Paralysis, tuberculosis, asthma	Blood is massaged externally on paralytic muscles and soup is prepared by boiling the meat in water.	Orally and Externally
11	*Murghi*	Chicken	*Gallus domesticus*	15	Meat, Egg, Fat	Weakness and cough	Meat extract (soup) is prepared by boiling the meat in water. Egg yolk and albumin is used in cough after boiling	Orally and Externally
12	*Hiran*	Black Buck	*Antilope cervicapra*	17	Meat, fat, horns	Asthma, tuberculosis, weakness, piles	Horns are burnt and ash is used meat is cooked and fat is used for piles.	Orally and Externally
13	*Khargosh*	Indian hare	*Lepus nigricollis*	26	Liver/Flesh	Asthma, tuberculosis	Liver and flesh of the this animal is roasted and eaten	Orally
14	*Gaey*	Zebu/Humped Cattle	*Bos taurus indicus*	29	Milk, Butter, Ghee, Gallbladder	Weakness, asthma, epilepsy, skin diseases	Ghee is obtained by melting butter. *Piper nigrum* is embedded in the Gall bladder for 41 days. After this piper is removed and dried.	Milk, Butter And Ghee orally and externally. Ghee is massaged on chest in asthmatic patients. *Piper nigrum* is orally given for the treatment of epilepsy.
15	*Oont*	Camel	*Camelus dromedarius*	32	Milk, Fat, Wool	Jaundice, skin diseases, rickets	Milk is orally given, Wool is burned and ash is applied on wounds. Fat is massaged in Rickets.	Milk is oral. Ash is applied externally on wounds and fat is massaged in rickets.
16	*Bakri*	Goat	*Capra hircus*	21	Milk and Meat	Tuberculosis, conjunctivitis	Milk and Meat extract (soup) is given for tuberculosis and Asthma, milk is dropped in eyes for the cure of infection.	Orally and Externally
17	*Bheer/Bhaid*	Sheep	*Ovis aries*	19	Milk, meat and wool	General and sexual debility and skin wounds. Given to newborn babies in diluted form	Meat is boiled in water and extract (soup) is made and wool is burnt and ash is made	Orally and Externally
18	*Bhains*	Buffalo	*Bubalus bubalis*	21	Milk,meat, butter, and ghee	General weakness and paralysis	Milk, butter, and meat are used as tonic while sometimes ghee is massaged on paralytic muscles.	Orally and externally
19	*Aurat*	Women	*Homo sapiens*	14	Milk	Meningitis	Milk is massaged on the cranium of the patients	Externally
20	*Gadha*	Donkey	*Equus asinus*	24	Milk	Asthma, tuberculosis	Milk is administered	Orally

## 4 DISCUSSION

In the present study, the data collected from nomadic pastoralists of Cholistan desert was examined critically and thoroughly, and only the information advocated, averred and asserted by the native people, quacks and practitioners of alternative medicines are presented. All the relevant information regarding the animal's local name, parts or products used to cure the different diseases has been provided by respondents and also the methods of preparation of these products. This study revealed 20 animal species that are utilized for medicinal purposes indicating very rich ethnomedicinal knowledge of the study area i‐e Cholistan desert of Bahawalpur. We found that among 20 animals nine animals are domestic while 11 are wild animals, from which nine are mammals, four birds, four reptiles and three insects. According to the survey, six animals and their products are used for the management of asthma, six are used for general weakness, five for sexual debility, five for tuberculosis, three for skin problems, three for paralysis, three for arthritis, two for cough, two for leucoderma, one for meningitis, one for impotency, one for piles, one for leprosy, one for diabetes, one for rickets, one for dropsy and one animal is used for the treatment of epilepsy. The study concerning with the parts of animals used by the people, milk of 6 animals, butter and ghee of two animals, wool of two animals, flesh/ meat of eight animals, fat of five animals is being used while three animals are used and horns of one animal is used as medicine. It was well reported in previous studies that animals including wild and domestic, and their organs/by‐products such as bones, hooves, feathers, skin and tusks are important components for the preparation of curative, protective, and preventive medicine in zootherapy (Anyinam, 1995; Kang, 2003; Kushwah et al., 2017; Soewu et al., 2012). Meyer‐Rochow (2017) and Jaroli et al. (2010) described that the nature of animal‐derived medicinal substances depends upon the ailment and disease to treat which included squeezing, direct use, crushing/grinding, burning, cooking, wrapping, powdering, and drying (Jaroli et al., 2010; Meyer‐Rochow, 2017). An egg is the product of animals and ostrich's egg (*Struthio camelus*) is used to cure muscle strain, broken bone, and paralysis (Kendie et al., 2018). It was further investigated that different types of human diseases like cough, asthma, paralysis, tuberculosis, earache, erectile dysfunction, herpes, weakness and muscular problems are treated with animals or by body parts or by‐products like organs, milk, flesh, blood, antler and feathers (Haileselasie, 2012).

Many of these animals are also used in another traditional system of medicine in Middle Eastern countries. Hooper and Field (1937) described that twelve different animals including cow, camel, sea sponge, honey, fish, sheep, silkworm and squid in Iraq are used as traditional medicine (Hooper & Field, 1937). A published study conducted in Egypt by a physician revealed that 41 drugs of animal origin out of 640 are present in the Cairo market (Meyerhof, 1918). During the 1970s, one study conducted in Pakistan stated that 31 organic substances were listed as animal parts and products that is 9% of all medicinal substances in the catalog of traditional medicines (Lev, 2003). It was reported in Ethiopia that the majority of human population (70%) and livestock (90%) uses traditional medicine to treat various ailments (Birhanu, 2013). Kendie et al. (2018) investigated the application of 51 animal species and their products to treat or to prevent about 36 different diseases (Kendie et al., 2018). Furthermore, it was revealed in a study in Degu tribes in Tigray region of Ethiopia that traditional medicine containing animals (23 animals) showed potential to cure ailments (Haileselasie, 2012) and about 16 animal species were identified and used for the cure of 18 different human ailments in the Kafta‐Humera District, Northern Ethiopia (Yirga et al., 2011). Whiting et al. (2010) in South Africa reported that there were 147 vertebrate species (60 mammals, 53 birds, 33 reptiles and one amphibian) used as traditional medicine for the treatment of various disorders (Whiting et al., 2010) while Oliveira et al. (2010) reported 23 animal species having applications in zootherapy (Oliveira et al., 2010). Our observations also coexisted with the study of Chakravorty et al. (2011) who reported the potential of 36 vertebrate species comprised of mammals (50%), reptiles, fishes, birds and amphibians for the treatment of various ailments (Chakravorty et al., 2011).

Further, it was reported that different people from different areas of the world used different parts/products of animals which contained medicinal substances for the treatment of different kinds of ailments (Alves & Rosa, 2005) and it was also reported by Meyer‐Rochow (2017) that selected organs of invertebrate animals are used as traditional medicines ( Meyer‐Rochow, 2017). Borah and Prasad (2017) explored that there were about 40 different ailments which could be treated using a total of 44 different species of animals (Borah & Brasad, 2017). Pieroni et al. (2004) carried out an ethnopharmacognostic survey through classical ethnographical and ethnobiological methods on the traditional pharmaceutical knowledge (TPhK) of natural remedies used for healing humans in Central Lucania, inland southern Italy. They recorded approximately 110 remedies of plant origin from 103 botanical taxa, 30 of animal origin and 20 industrial products or minerals. Their study data suggested the new inputs for pharmacological and phytochemical studies among Mediterranean folk pharmacopoeias and to sustain environmentally integrated projects focusing the maintenance of TPhK through controlled gathering activities or breeding of local medicinal species (Pieroni et al., 2004). Another study conducted by Quave et al. (2010) on the comparative assessment of zootherapeutic remedies through interviewing the informants regarding zootherapeutic traditions of Albania, Italy, Nepal and Spain. They identified 80 species used in zootherapeutic remedies ranked by consensus indices, representing four phyla in the animal kingdom i‐e Arthropoda, Annelida, Mollusca and Chordata showing that the selection of medicinal fauna is mediated by human subsistence patterns (Quave et al., 2010). Gonzalez and Villalobos (2014) conducted a review on research reports published during 1881 to 2012 synthesizing the traditional knowledge related to the use of wild vertebrates in folk medicine of the central western sector of Spain. They documented 182 traditional remedies related to 18 diseased groups and highlighted the species richness useful to treat the infectious and parasitic diseases (Gonzalez & Villalobos, 2014).

## 5 CONCLUSION

As current investigations are majorly based on the deep discussions with nomads of Cholistan desert and their knowledge in the treatment of various diseases and maintain general well‐being. The results of this study explored that parts of animals, raw material and or their products occupied key positions in the traditional medicine and medical practices for the treatment of a vast range of different ailments. It could be concluded from the current investigations that traditional people mostly depend on spiritual as well as zootherapy due to the absence of modern medical facilities, lack of awareness, expensive drugs and unavailability of proper transportation. Based on this communication, we could recommend that neglected knowledge of this type should be included in conservation strategies and management of faunistic assets. However, it was also found that some people from local areas of Cholistan now refrained to sacrificing animals due to strong material and spiritual ties with their animals. Furthermore, to overcome the diseases in these areas more research is needed for experimental confirmation. Moreover, significantly vital indigenous knowledge is getting lost together with the experts and elders due to scarce efforts to conserve, manage and proper documentation of indigenous knowledge and skills in the field of zootherapy. So, along with proper documentation and management of the indigenous knowledge regarding ethnozoological, more research should be done to test the animal's products to explore their bio‐active components in the raw material of these animals scientifically for product development.

## CONFLICT OF INTEREST

Authors declare that there is no conflict of interest.

## AUTHOR CONTRIBUTION

**Saeed Ahmad:** Conceptualization; Resources. **Rabia Zahid:** Data curation; Formal analysis. **Muhammad Riaz:** Investigation; Methodology. **Naveed Munir:** Resources; Software. **Imtiaz Mahmood Tahir:** Resources; Software. **Hina Anwar:** Validation. **Muhammad Daniyal:** Visualization. **Faiza Jabeen:** Writing‐original draft. **Ejaz Asharf:** Writing‐review & editing. **Ghulam Sarwar:** Methodology; Writing‐original draft. **Ghulam Rasool:** Supervision; Validation.

### PEER REVIEW

The peer review history for this article is available at https://publons.com/publon/10.1002/vms3.491.

## Data Availability

The datasets generated during and/or analysed during the current study are available from the corresponding author on reasonable request.
